# Modeling and Simulation in Capacity Degradation and Control of All-Solid-State Lithium Battery Based on Time-Aging Polymer Electrolyte

**DOI:** 10.3390/polym13081206

**Published:** 2021-04-08

**Authors:** Xuansen Fang, Yaolong He, Xiaomin Fan, Dan Zhang, Hongjiu Hu

**Affiliations:** 1 Shanghai Institute of Applied Mathematics and Mechanics, School of Mechanics and Engineering Science, Shanghai University, Shanghai 200072, China; friendship2000@163.com (X.F.); yaolonghe@shu.edu.cn (Y.H.); Fan_xm26@163.com (X.F.); 2 Shanghai Key Laboratory of Mechanics in Energy Engineering, Shanghai 200072, China; 3 School of Mechatronic Engineering and Automation, Shanghai University, Shanghai 200072, China; dan.zhang@shu.edu.cn

**Keywords:** solid polymer electrolyte, time-aging, capacity degradation, all-solid-state battery

## Abstract

The prediction of electrochemical performance is the basis for long-term service of all-solid-state-battery (ASSB) regarding the time-aging of solid polymer electrolytes. To get insight into the influence mechanism of electrolyte aging on cell fading, we have established a continuum model for quantitatively analyzing the capacity evolution of the lithium battery during the time-aging process. The simulations have unveiled the phenomenon of electrolyte-aging-induced capacity degradation. The effects of discharge rate, operating temperature, and lithium-salt concentration in the electrolyte, as well as the electrolyte thickness, have also been explored in detail. The results have shown that capacity loss of ASSB is controlled by the decrease in the contact area of the electrolyte/electrode interface at the initial aging stage and is subsequently dominated by the mobilities of lithium-ion across the aging electrolyte. Moreover, reducing the discharge rate or increasing the operating temperature can weaken this cell deterioration. Besides, the thinner electrolyte film with acceptable lithium salt content benefits the durability of the ASSB. It has also been found that the negative effect of the aging electrolytes can be relieved if the electrolyte conductivity is kept being above a critical value under the storage and using conditions.

## 1. Introduction

Solid polymer electrolytes (SPEs) are emerging as a promising solution to achieve broad electrochemical stability window, excellent mechanical properties, and good safety for developing high-performance all-solid-state batteries (ASSB) [1,2,3,4]. Due to the effects of the preparation process and electrochemical operation, many SPEs are usually in a non-equilibrium state, in which the free volume and microstructure would evolve with time [5,6]. That is to say, the time-aging may occur in the amorphous polymer and can significantly affect the migration of lithium-ion in the solid electrolyte [7,8]. Subsequently, it would change the distribution of lithium concentration in the active material and lead to cell capacity variation. Consequently, the evolution of ionic conduction of SPEs and relevance to electrochemical behavior of ASSB during the aging process of polymer electrolyte are crucial scientific problems to be solved.

Knowledge of the time-aging properties of SPEs is very pivotal to understand the long-term performance of ASSB for reliable electrochemical device applications. As one of the most critical parameters of SPE, the time-dependent conductivity of polymer electrolyte materials has already attracted much attention. As early as 2003, Kumar et al. noticed that storage time had greatly affected the ionic conductivity of poly(ethylene oxide) (PEO) based SPE with lithium perchlorate (LiClO_4_) in the application temperature (0 to 68 °C) [7]. They show that due to a reduction in the size of the coordinating sphere around the lithium-ion, physical aging enhances the conductive performance of such a composite electrolyte. Nevertheless, in 2015, Lasinska et al. delved deeply into the evolution of physical properties of SPE based on poly(acrylonitrile-*co*-butyl acrylate) with different lithium salt content and storage time under argon atmosphere [8]. It was revealed that the conductivity of aged electrolytes weakened remarkably during the physical aging process, which affected the continuity of conductivity pathways ground on ion–ion interactions. Recently, Sengwa et al. studied the dependence of time-aging on the electrical and structural properties of lithium triflate (LiCF_3_SO_3_)-doped polymer blend matrix of PEO and poly(methyl methacrylate) (PMMA) [5]. The ionic conductivity of the SPE film was found to initially increase significantly, and then drop by more than one order of magnitude as the aging time rises from the day first to the one-year. These different results indicate that the time-aging appears to act upon a complicated role on the lithium-ion diffusion in solid electrolytes. It is still an open question whether the ion transport in the aging SPE mainly depends upon the relaxation of polymer cooperative chain segmental motion or not. However, one may reach a consensus that adding a small number of inorganic nanofillers such as Al_2_O_3_ [7], montmorillonite (MMT) clay [5,9], and LiAlO_2_ [10] into the polymer matrix can affect the conductivity of aging electrolyte materials. Meanwhile, the aging rate would be influenced by the nature and loading of ionic salt as well as different types of mixed salts. Moreover, it also correlates with the processing parameter, storage temperature, and even mechanical history of SPE.

In addition to the performance of the solid electrolyte itself, interfacial stability between solid electrolyte and electrode is also necessary for aging-resistant secondary lithium polymer batteries [11,12]. It is highly significant to study the transport and interfacial properties of SPEs in lithium cells during calendar aging. The published works have focused on suppressing the reactivity of lithium salts (LiX) in PEO-based SPE with a lithium metal anode to control the increment of passivation layer resistance in the process of storage period [10]. It was found that the addition of nano-size TiO_2_, Al_2_O_3_, or SiO_2_ particles could substantially not only enhance the ionic conductivity of PEO with LiClO_4_ at ambient temperatures but also boost the interfacial stability, which endowed the lithium batteries with better resistance to physical aging. Besides, the dispersion of ferroelectric microparticles (BaTiO_3_, LiNbO_3_, PbTiO_3_ [11] or g-LiAlO_2_ [13,14]) into the PEO-LiX electrolyte was verified as a remarkably effective method to stabilize the impedance of the SPE/electrode interface. Li_3_PO_4_ thin-film coating on LiCoO_2_ can act as the oxidation barrier for ethylene oxide-based SPE, contributing to the improvement of the interface degradation between SPE and the cathode [15]. Moreover, the SPE membrane with semi-interpenetrating polymer networks (s-IPN) structure exhibited eminent stability toward lithium metal [16]. Noticeably, the interfacial resistance is related to not only the formation of the lithium passivation layer but also the variation in the contact area of electrolyte and battery electrodes. Tian and Qi have found that contact loss formed during cell fabrication resulted in degradation of the battery performance [17]. The imperfect contact may be worsened during the physical aging process, where excess free volumes progressively escape from the polymer-lithium salt system due to a naturally occurring densification of the polymer structure [18,19,20]. Although the physical aging-induced SPE volume decrease may reduce the contact area of electrolyte/electrode interface and correspond to the electrical properties, up to now, no previous studies have paid close attention to the effect of time-aging on the interfacial contact between the solid electrolyte and electrode.

It is crystal clear that the capacity fades of ASSB can be caused by many factors, including the deterioration of the physical-chemical properties of the electrode, the relaxation of solid electrolytes, as well as the increase in the interface impedance between the cell components. Grillon et al. reported that the capacity loss of the all-solid-state thin-film micro-battery (Li/LiPON/LiCoO_2_) was induced by the diminution of the inserted charges number into the cathode due to some structural changes in LiCoO_2_ material [21] and the growth of cathode electrolyte interfacial layer [22]. Meanwhile, extensive studies have been accomplished for illustrating the influence of electrochemical temperature, current rate, state-of-charge (SOC), and depth-of-discharge (DOD) on the aging behavior of lithium-ion batteries with inorganic solid electrolytes. Danilov et al. discovered that the capacity of ASSB would decrease at a high discharge rate [23]. Grillon et al. found that the capacity decay for a micro-battery system increased with the cycling temperature rising, and the aging effect was dependent upon the DOD and discharge current [24]. However, owing to the codependency of aging mechanisms, very few literature have documented the effect of the electrochemical operation on the degradation of SPE based cell. The prediction of the aging-dependent capacity for this kind of ASSB under various storage and usage conditions is still a critical and challenging goal. The theoretical model and simulation for deriving optimized designs on ASSB with aging SPE are still somewhat lacking.

In this manuscript, focusing on the polymer electrolyte during the physical aging process, we will develop a continuum model consisting of electrochemistry, mechanics, and thermodynamics for quantitatively describing the electrochemical performance of ASSB in service. The capacity degradation induced by time-aging SPE will be unveiled for the first time by a finite difference method. Meanwhile, we will also systematically explore the aging trend of the cell with different operation conditions (temperature and discharge rate), lithium salt concentration of the electrolyte, and the thickness ratio of electrolyte relative to the electrode. The primary aim is to reveal the effect mechanism of aging SPE on the long-term life of the secondary batteries and to provide theoretical support for the optimal design and utilization of advanced ASSB.

## 2. Methodology

### 2.1. Basic Electro-Chemical Model

**Figure 1:**
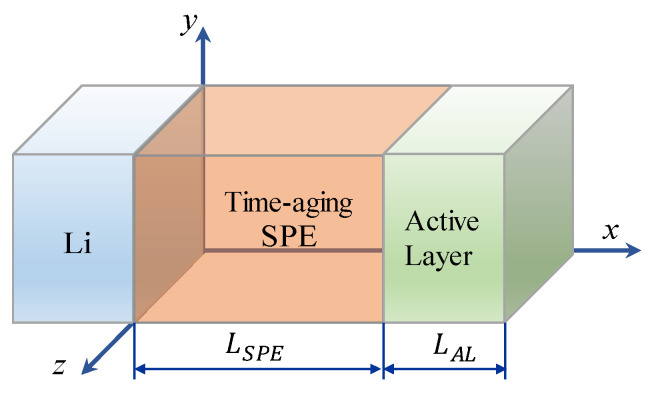
Schematic of an all-solid-state battery with time-aging solid polymer electrolytes (SPE).

To demonstrate the method, here we consider a typical layered ASSB as illustrated in Figure 1. It contains a Li metal negative electrode, a time-aging SPE, and a LiCoO_2_ positive electrode. For this multi-field system, it is known that to establish the basic governing equation, one can start from two aspects: the electrochemical reaction at the SPE/electrode interface and the transports of cations and anions under the electric field in SPE and LiCoO_2_. For the former, according to Qi et al. [17], it can be described by the Butler-Volmer equation as follows:

(1) $$\;I_{i}=I_{i}^{0}\left\{exp\left(\alpha_{i}\frac{F}{RT}\eta_{i}^{over}\right)-exp\left[-\left(1-\alpha_{i}\right)\frac{F}{RT}\eta_{i}^{over}\right]\right\},\;i=Li\;or\;LiCoO_{2}$$

Here, $I_{i}$ and $I_{i}^{0}$ are the partial anodic/cathodic current at the electrode surface and the related exchange current, respectively. To simplify the description, $I_{i}^{0}$ is detailed in Appendix A. $\alpha_{i}$, F, and $\eta_{i}^{over}$ are the charge transfer coefficient, Faraday constant, and the over potential, respectively. *R* is the molar gas constant, and *T* represents the temperature.

For the latter, in terms of the Nernst-Planck theory, the migration of cations and anions in SPE in response to the electric field induced by the above electrochemical reactions can be expressed as follows [25]:

(2) $$\partial c_{j}/\partial t+\nabla \cdot h_{j}=0$$

where $c_{j}$ is the concentration of cations ($j=Li^{+}$) or anions ($j=X^{-}$), $h_{j}$ is the flux which is derived from the generalized Nernst-Planck formula as

(3) $$h_{j}\left(x,t\right)=-D_{j}\nabla c_{j}\left(x,t\right)-\frac{\xi_{j}F}{RT}D_{j}c_{j}\left(x,t\right)\nabla \phi_{SPE}\left(x,t\right)-\frac{\Omega_{j}}{RT}D_{j}c_{j}\left(x,t\right)\nabla p$$

where $D_{j}$ is the diffusivity coefficient of species *j* in the SPE, and $\xi_{j}$ is the valence (dimensionless), $\Omega_{j}$ is the related partial molar volume, and $p=-Ktr\varepsilon +K\sum_{j=Li^{+},X^{-}}\Omega_{j}\left(c_{j}-c_{j}^{0}\right)$ is the hydrostatic pressure, which can be obtained by solving a set of elastic mechanics Equations as detailed in the Appendix B. It can be seen that there are three variables to be determined in Equation (3). It can be seen that there are three variables to be defined in Equation (2), i.e., $c_{j}$ and $\phi_{SPE}$, but there are only two Equations. Therefore, to determine the remaining quantity, the so-called neutral condition is also introduced according to Grazioli et al. [26]:

(4) ∇⋅I=0

where $I=F\sum_{j=Li^{+},X^{-}}\xi_{j}h_{j}\left(x,t\right)$ is the electric current density.

As for the solid migration of lithium in the active layer, the influence of the electric field will be minimal, and the governing Equation can hence be written as [27]:

(5) $$\frac{\partial c_{Li}\left(x,t\right)}{\partial t}+\nabla \cdot j_{Li}=0,\;j_{Li}=-D_{Li}\nabla C_{Li}\left(x,t\right)$$

where $C_{Li}\left(x,t\right)$ and $D_{Li}$ represent the concentration and diffusion coefficient of Li, respectively.

### 2.2. Discharging Capacity of a Cell with Time-Aging SPE

Usually, when discharging an ASSB at constant current, the lithium inserted in active cathode materials would gradually increase, leading to the decreased cell voltage. The related discharging capacity of this cell is then defined as the output when the discharge voltage reaches the cutoff value, which is about 3.8 V for LiCoO_2_. Since the cell is under constant-current operation upon this process, the discharging capacity $Q_{out}$ can then be estimated by:

(6) $$Q_{out}=i_{n}\times A\times t_{c}$$

where $i_{n}$ is the operation current, which is usually estimated by $i_{n}=i_{1C}\times C_{rate}$, in which $i_{1C}$ is the current at 1C rate, and $C_{rate}$ represents the operation C-rate. A and $t_{c}$ are the effective contact area and cutoff time, respectively.

For an ASSB with time-aging SPE, as the essence of the time-aging of SPE is the process of free volume escaping outward [18,19,20], it can be expected that the time-aging would result in not only the decline of ion migration in the electrolyte but also the decrease in bulk volume of SPE because of the polymer densification induced by the aging process. As a result, changes in cutoff time and contact area caused by aging may affect the electrochemical performance of the ASSB. In this case, since the aging time is much longer than the discharge time, as described in Section 2.3, Equation (6) still holds, but the cutoff time $t_{c}$ and effective contact area *A* within it will be affected by aging.

To identify the decline of ion migration in the electrolyte caused by time-aging, the electrochemical impedance spectrometer (EIS) can be adopted to test the impedance spectra of SPE under the time-aging and analyze the changes of ionic conductivity (k). Then the ionic mobility (µ) is calculated on the basis of the Einstein relation [28]:

(7) $$µ=\frac{k}{nq}$$

where *n* is the number of ions per unit volume, and *q* is the charge carried by ions. Thereafter, since the mean diffusion coefficient of ion (D) is a function of the ionic mobility according to the Einstein relation, i.e., $D=\mu k_{B}T/q$, the relationship between mean diffusion coefficient D and ionic conductivity k can be obtained by:

(8) $$D=\frac{k_{B}T}{nq^{2}}k$$

were $k_{B}$ is the Boltzmann constant, and T is the absolute temperature. The magnitude of D may be defined by the harmonic mean of the ionic diffusion coefficients of Li^+^ cation ($D_{Li^{+}}$) and X^−^ anions ($D_{X^{-}}$), where a binary salt (LiX) is assumed to fully dissociate in the polymer electrolyte in two ionic species [26].

To further estimate the effective contact area under the time-aging process, we assume that SPE is a cuboid isotropic homogeneous material. The relative change in contact area upon aging is mainly caused by the volume variation induced by the reduction of free volume, and it can be acquired by:

(9) $$\frac{A}{A_{0}}={\left(\frac{V}{V_{0}}\right)}^{\frac{2}{3}}={\left(\frac{\lambda V_{f_{0}}+V_{s}}{V_{f_{0}}+V_{s}}\right)}^{\frac{2}{3}}={\left[1-\left(1-\lambda \right)v_{f}^{0}\right]}^{\frac{2}{3}}$$

where $A_{0}$ and $V_{0}$ represent the contact area between electrolyte and electrode, and the bulk volume at the initial aging time ($t_{a}=0$), respectively. *A* and V are the related area and volume at the time $t_{a}$. λ is a dimensionless aging-related coefficient. $v_{f}=V_{f}/V$ is the volume content of the free volume $V_{f}$ relative to the total volume V which is composed of the solid volume $V_{s}$ occupied by the polymer segment and the free volume $V_{f}$. 0 in the subscript/superscript represents the aging time ($t_{a}=0$). Since the relationship between ionic diffusion coefficient of SPE and free volume ($v_{f}$) can be written as [29] $D=Me^{-\alpha /v_{f}}$, where M and α are the material constants, the volume content at time $t_{a}$ would be:

(10) $$v_{f}=\frac{lnD^{0}-lnM}{lnD-lnM}v_{f}^{0}$$

Then, the dimensionless coefficient λ can be obtained by the correlation between the aforementioned volume parameters and written as:

(11) $$\lambda =\frac{1-v_{f}^{0}}{1-v_{f}}\frac{v_{f}}{v_{f}^{0}}$$

To sum up, combining Equations (6) and (9), one can achieve the capacity of ASSB at constant-current discharge during the time-aging SPE as follows.

(12) $$Q_{out}={\left(1-\left(1-\lambda \right)v_{f}^{0}\right)}^{\frac{2}{3}}{\bar{t}}_{c}\times Q_{out}^{0}$$

where $\;Q_{out}^{0}=i_{n}A_{0}t_{c}^{0}$ is the discharge capacity at $t_{a}=0$, ${\bar{t}}_{c}=t_{c}/t_{c}^{0}$ is the dimensionless cutoff time.

### 2.3. Solving Conditions and Model Parameters

**Figure 2:**
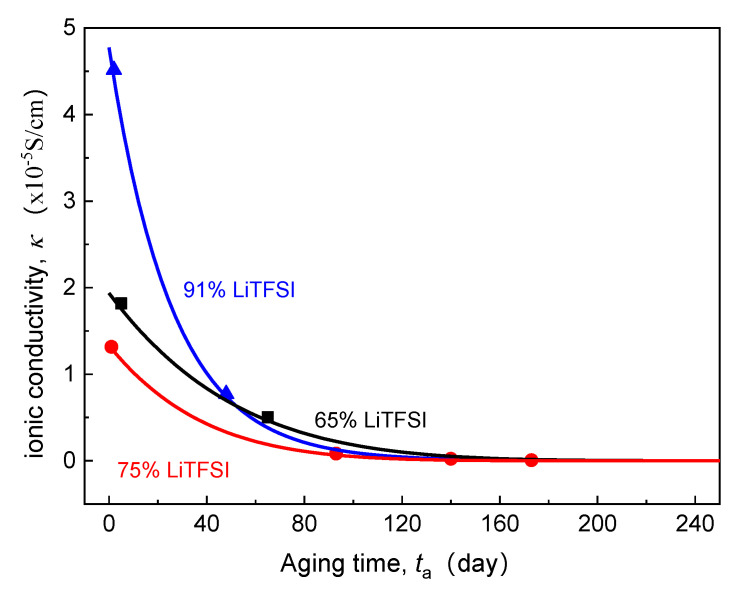
Effect of aging time on ionic conductivity of SPE with different lithium salt contents (20 °C).

Section 2.1, Section 2.2 and Section 2.3 describe the coupled multi-field Equations for the cell capacity degradation induced by time-aging SPE. The model consists of a series of electrochemical and mechanical PDEs. By applying proper initial and boundary conditions, these nonlinear PDEs can be numerically solved using a finite difference method software COMSOL Multiphysics^®^. Previously, Lasinska et al. [8] presented their excellent experimental work on the evolution of ionic conductivity of the electrolyte based on LiTFSI and poly(AN-co-BuA) against the aging time of nearly one year. The effects of time-aging and lithium salt content on the conductivity of SPE are as shown in Figure 2. In this research, we have taken this reported LiTFSI/poly(AN-co-BuA) as the solid electrolyte to conduct the computational simulations and numerically optimize the capacity degradation of Li/SPE/LiCoO_2_ induced by the time-aging SPE according to the above-stated continuum model. Since the governing equations as shown in Equations (4), (5) and (8) are fully coupled, analytical solutions are difficult to obtain. Therefore, the numerical difference method is employed here to solve the problem. Since the cell is composed of three parallel layers, as illustrated in Figure 1, the problem is hence simplified to a 1D model. Table 1 summarizes the corresponding initial conditions and boundary conditions for the problem. Other parameters used in the simulations are listed in Table 2.

## 3. Results and Discussion

### 3.1. Dependency of Cell Discharging Capacity on Time-Aging SPE

**Figure 3:**
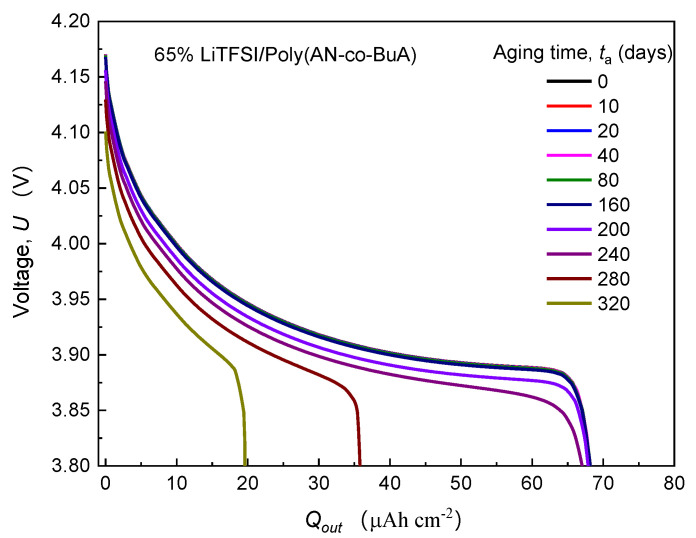
Effect of physical aging on the discharge curve ($U-Q_{out}$) at 1C rate.

First of all, the evolution of cell voltage with the discharge capacity for various time-aging SPEs was tracked to demonstrate the dependency of aging on the cell capacity. Taking the typical 65%LiTFSI/poly(AN-co-BuA) as an example, Figure 3 shows the discharge curve ($U-Q_{out}$) of ASSB concerning this SPE at the different aging times (0–320 days). For other aging electrolytes with lithium salt loading of 75% and 91%, the cells exhibit a similar degradation tendency.

**Figure 4:**
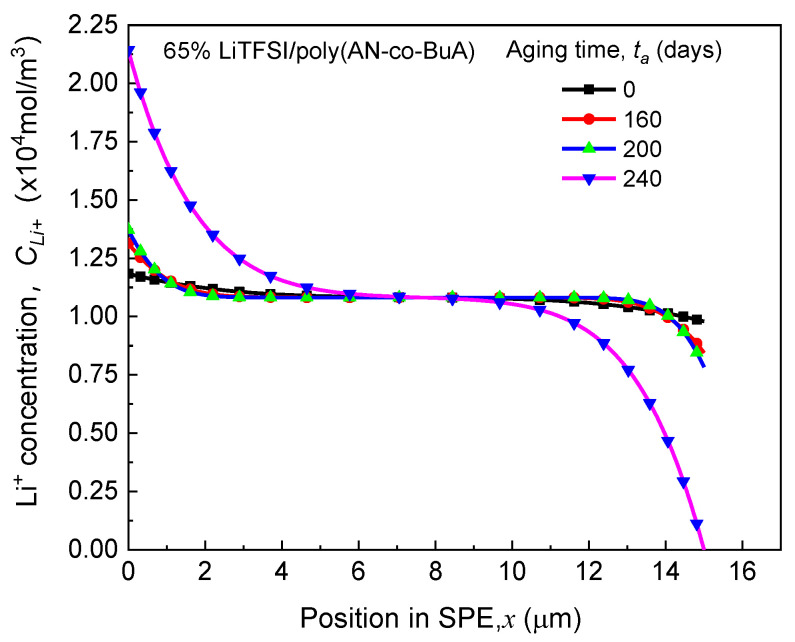
Effects of aging time on Li^+^ concentration distribution in SPE at the cutoff time.

It can be observed from Figure 3 that the discharge curve at the initial time-aging stage ($t_{a}=0$) almost coincides entirely with those of the following 10, 20, 40, 80, and 160 days, and the values of $Q_{out}$ before 240 days are all around 68 Ahcm^−2^. Therefore, the ASSB with 65% LiTFSI/poly(AN-co-BuA) still has good resistance to the time-aging after storage of more than half a year. In other words, the aging effect of the electrolyte on the discharge capacity of the ASSB is slight during this period. As aging time ($t_{a}$) increases, however, the $U-Q_{out}$ curves drop steeply. When $t_{a}$ is 280 days and 320 days, the discharge capacities are 36 Ahcm^−2^ and 20 Ahcm^−2^, respectively, which have been significantly reduced by 47% and 70% compared with its standard capacity (68 Ahcm^−2^). The main reason is that the integrality of ion transport in the cell system has been damaged due to electrolyte aging. To show this reason, the distribution of lithium-ion concentration in SPE discharged at the cutoff time was extracted further, as shown in Figure 4.

**Figure 5:**
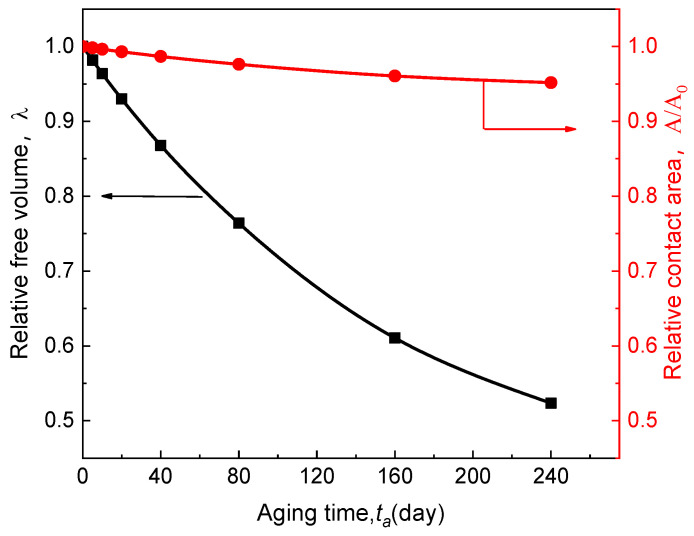
Effect of time-aging on free volume in SPE and its contact area with the electrode.

(1) As can be seen from the ASSB structure (see Figure 1) above, the left-side (anode) and right-side (cathode) are corresponding to the outflow and insertion of Li^+^, respectively, under the discharging, which means that ions move from left to right in the SPE. It can be anticipated that the higher the ion mobility, the smaller the difference in concentration will be observed between the two sides. Conversely, a tremendous difference in ion concentration between the two sides means worse ion mobility. Therefore, Figure 4 indicates that the transport performance of ions continues to deteriorate with aging. When the aging time is relatively short (200 days), though the mobile rate of the cation has decreased, it still matches with the lithiation requirement of the cathode at the right-side of the electrolyte, and thus, the discharge capacity has not changed significantly, as shown in Figure 3. However, after 240 days of aging, when the diffusion coefficient of lithium-ion in SPE declines to a threshold that the rate of Li^+^ passing through the SPE can no longer meet the consumption of lithium embedding in the cathode, the Li^+^ concentration on the right-side of SPE would rapidly reduce to 0. This will interrupt the electrochemical reaction of the electrode, shorten the discharge time, and thereby decrease the discharge capacity of the ASSB significantly.

(2) During the time-aging of SPE, the structural relaxation induced by the lattice contraction [31] and free volume diffusion in the solid electrolyte also cause the reduction of effective contact between the SPE and LiCoO_2_. The related influence can be obtained by extracting the variation of the aging-related parameters λ and $A/A_{0}$. These results are shown in Figure 5.

In Figure 5, it can be seen that the parameter λ reduces rapidly with the aging time, indicating that free volume within SPE decreases upon aging. Affected by this, the effective contact area A between SPE and electrode is also steadily decreasing. When the aging time reaches 240 days, A is reduced by nearly 5%. This shows that in addition to the decline in ion conductivity of time-aging SPE, the reduction in the effective contact area is also an essential factor for the decrease of ASSB.

**Figure 6:**
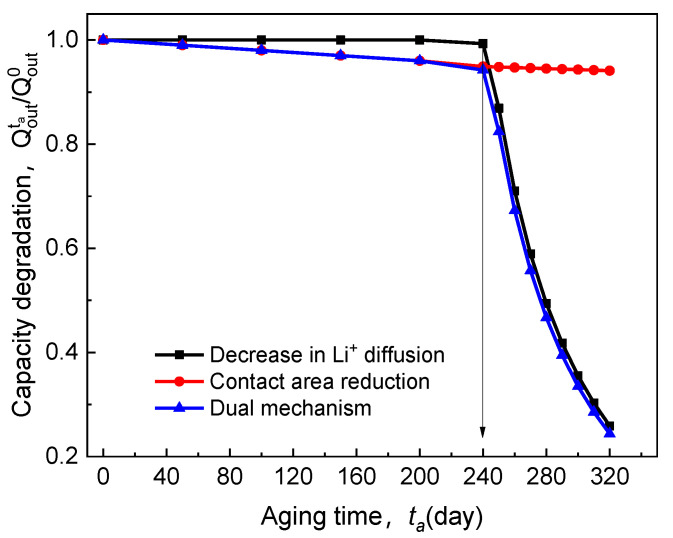
Effect of electrolyte time-aging on discharge capacity degradation.

There are three aspects to explain the effect mechanism of electrolyte time-aging on the electrochemical performance of ASSB, as shown in Figure 6. In the figure, the red line presents how the capacity decay ($Q_{out}^{t_{a}}/Q_{out}^{0}$) caused by the decrease of the electrolyte-cathode interface contact area evolves with the aging time. The black curve represents the variation in $Q_{out}^{t_{a}}/Q_{out}^{0}$ resulted from the weakening of ion diffusion through the electrolyte material. Furthermore, the blue line is a consequence of the coupling of the former two. It can be observed that there is an obvious inflection point in the blue curve, and the corresponding critical aging time ($t_{a}^{cr}$) is 240 days. Before this, $Q_{out}^{t_{a}}/Q_{out}^{0}$ falls off slowly, controlled by the loss of contact area, and the discharge capacity can still be maintained at over 90%. The transport of Li^+^ relies on its interaction with the segmental movement of macromolecules [32,33]. That is, ions penetrate through the electrolyte via the interaction of complexation and dissociation of the polar groups on the side chains. Thus, with the increase of aging time (>240 days ), a large amount of free volume in the electrolyte diffuses outward; the rotation of the C–C bond in structural elements of the electrolyte is limited, and the migration of Li^+^ would be dramatically diminished. Then, in this stage, ion diffusion degradation is the primary cause of cell discharge failure. It can be verified that the blue curve and the black curve in Figure 6, almost overlap with each other after their inflection points. Importantly, Figure 6 clearly shows that such SPE-based ASSB has a service life of less than one year (≤240 days) as the cell capacity experiences a sharp drop. That is to say, the engineering applications of the ASSB with Li/65% LiTFSI/poly(AN-co-BuA)/LiCoO_2_ will confront severe challenges in this situation. Therefore, it is an urgent task to delay the aging progress of ASSB and increase its cycle life through the optimal design of service conditions and electrolyte materials. To this end, the following three sections will illustrate the influence of discharge rate, operating temperature, the lithium salt concentration, and relative thickness of the electrolyte on the capacity loss of ASSB during the time-aging of SPE.

### 3.2. Effect of Discharge Rate and Operation Temperature

**Figure 7:**
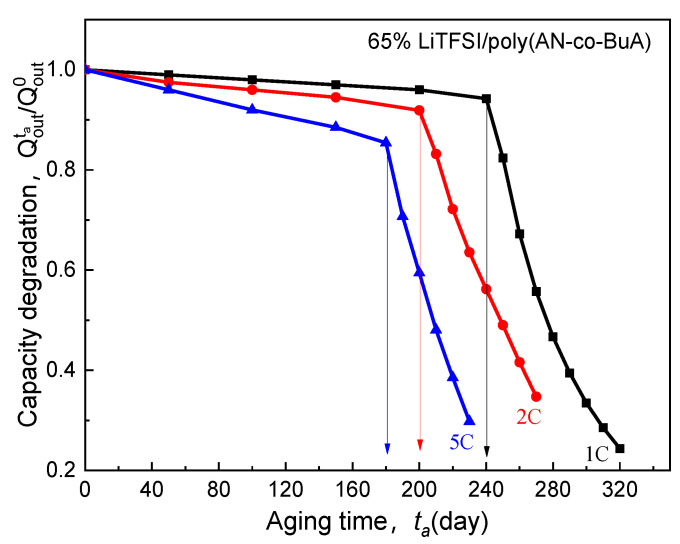
Effect of discharge rate on electrolyte-aging induced capacity degradation.

Figure 7 shows the influence of lithiation rate on the electrolyte-aging induced capacity degradation (EICD). In the simulations, the $C_{rate}$ range is 1C to 5C. The temperature is considered to be 20 °C, and the temperature rise caused by the high current is not taken into account. This is because an ASSB composed of Li/65% LiTFSI/poly(AN-co-BuA)/LiCoO_2_ usually discharges at the constant current with the different rate from 1C to 5C under room-temperature (20 °C) environment. There is evidence that thin-film ASSB with the nickel-based current collect for the cathode and a lithium metal anode has good heat dissipation, which exhibits a thermal conductivity as high as 90.9 W/(m K) and 84.8 W/(m K), respectively [34].

**Figure 8:**
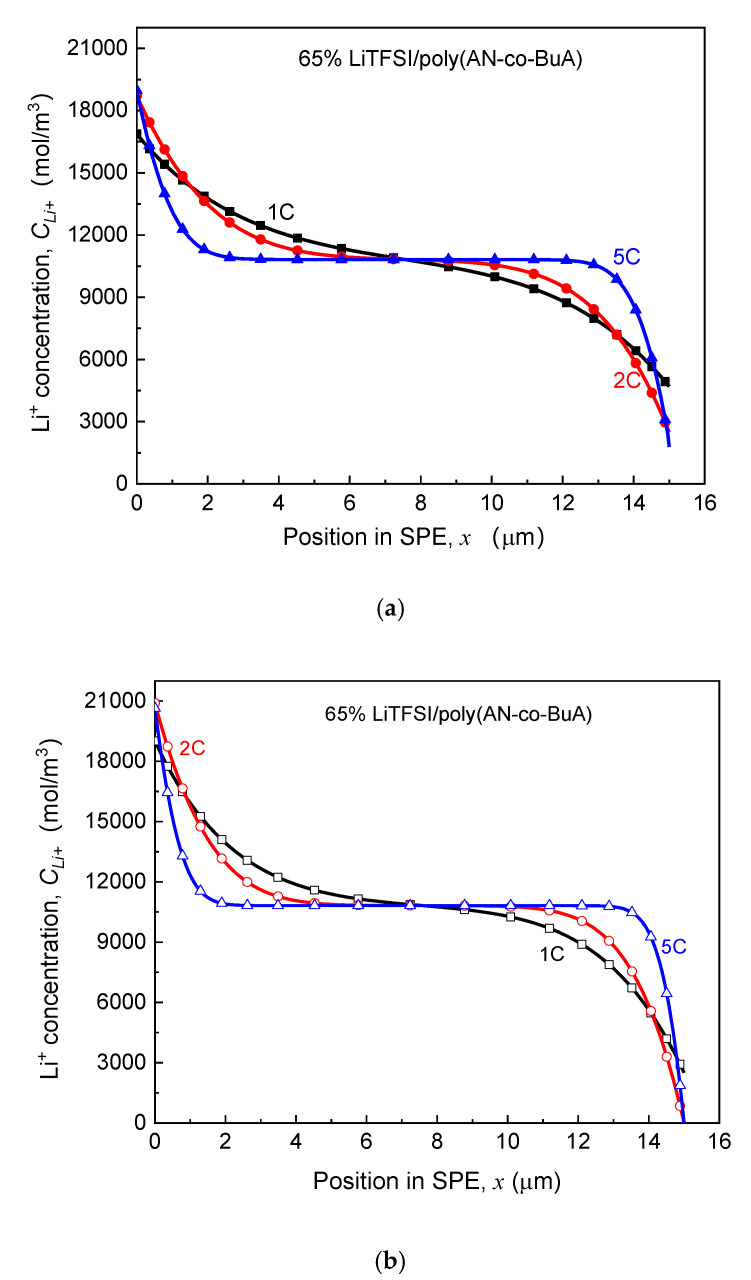

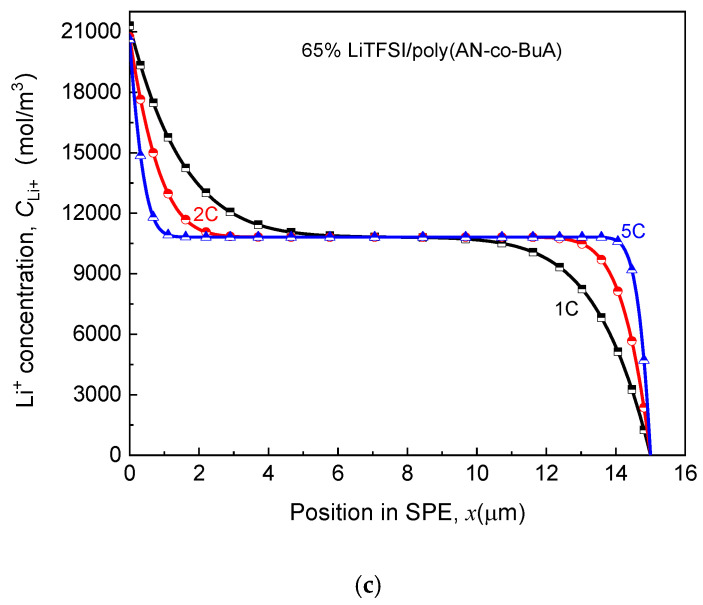
Effect of discharge rate on Li^+^ concentration distribution in SPE under different aging processes. (**a**) Aging time *t_a_* = 160 days; (**b**) Aging time ta = 200 days; (**c**) Aging time *t_a_* = 240 days.

As seen in Figure 7, for the electrolyte aged in the initial stage ($t_{a}\leq 40$ days), despite the higher discharge rate, the capacity degradation vs. aging time curve ($Q_{out}^{t_{a}}/Q_{out}^{0}$-$t_{a}$) of the ASSB discharged at 5C is nearly consistent with those at 1C and 2C, and the loss of discharge capacity is less than 2%. With the increase of aging time, the EICD effect gradually emerges during the high-rate discharge (5C). When $t_{a}=180$ days, the cell capacity decreases by 18%, and there is a drop inflection point of the $Q_{out}^{t_{a}}/Q_{out}^{0}$-$t_{a}$ curve. Following that, the electrochemical performance would deeply fall for the ASSB at a discharge rate of 5C. Also, the variation tendency of capacity with the aging time remains almost the same between the discharge rates of 1C and 2C before the aging time reaches 200 days. After that, it is found that the capacity of the ASBB discharged at the rate of 2C degrades rapidly. In contrast, the capacity of the ASSB with a lower lithiation rate of 1C is not subjected to an abrupt decline until the aging time reaches 240 days. Therefore, the discharge rate is a crucial factor to control EICD, and a high-speed C-rate will lead to a dramatic shortening of the cycle life of the ASSB. The reason behind it is that the cathode would consume lithium ions rapidly at a higher rate, which requires a faster ion-diffusion across the solid electrolyte. In contrast, the time-aging process of the SPE runs counter to this goal. Figure 8 is prepared to show this, which demonstrates the effect of different discharge rates (1-5C) on the distributions of Li^+^ concentration in the time-aging SPE (160 days, 200 days, and 240 days) for the cell at cutoff time.

According to the kinetic electrochemical equations of the electrode, as shown in Equation (1) and Appendix A, it can be achieved that the Li^+^ input (the left side that is adjacent to the lithium foil) and consumption (the right side that is adjacent to the LiCoO_2_) at the cathode increases with the rise of discharge rate. Focusing on the consuming side on the right, which directly affects the cutoff time, it can be found in Figure 8a that when the aging time of SPE is 160 days, the Li^+^ concentration declines with increasing the discharge rate due to the rapid consumption. Specifically, the Li^+^ concentration is 4700 mol/m^3^, 2491 mol/m^3^, and 1993 mol/m^3^ for 1C, 2C, and 5C at cutoff time, respectively. It is known that lithium concentration at the electrolyte/cathode interface required to maintain the normal lithiation reaction is about 2000 mol/m^3^. As such, at the discharge rate of 1C and 2C, the ion transport in the SPE can still guarantee the regular migration amount of Li^+^, and the discharge capacities of ASSB have not shown apparent changes at this time, as shown in Figure 7. Under the rate condition of 5C, however, the cell capacity has already presented a decline. When $t_{a}=200$ days, the ion concentration at the right-end of the electrolyte approaches 0 at the higher discharge rates (2C and 5C). In other words, the transport rate of lithium ions in the electrolyte has been unlikely to satisfy the requirements of the lithiation reaction of a positive electrode. Therefore, the cell capacity at the 2C discharge rate drops sharply at this point ($t_{a}=200$), while that of 5C is even worse, with the value of $Q_{out}^{t_{a}}/Q_{out}^{0}$ having decreased by nearly 50%, as in Figure 7. Moreover, when the aging time reaches 240 days, the values of Li^+^ concentration in the electrolyte side close to the cathode for three discharge rates decrease to 0. It means that the electrochemical performance at a low discharge rate (1C) is also about to deteriorate promptly. Furthermore, discharging at a relatively high rate will further intensify the negative influence of solid electrolyte time-aging, resulting in the long-term service performance of ASSB prematurely degraded.

**Figure 9:**
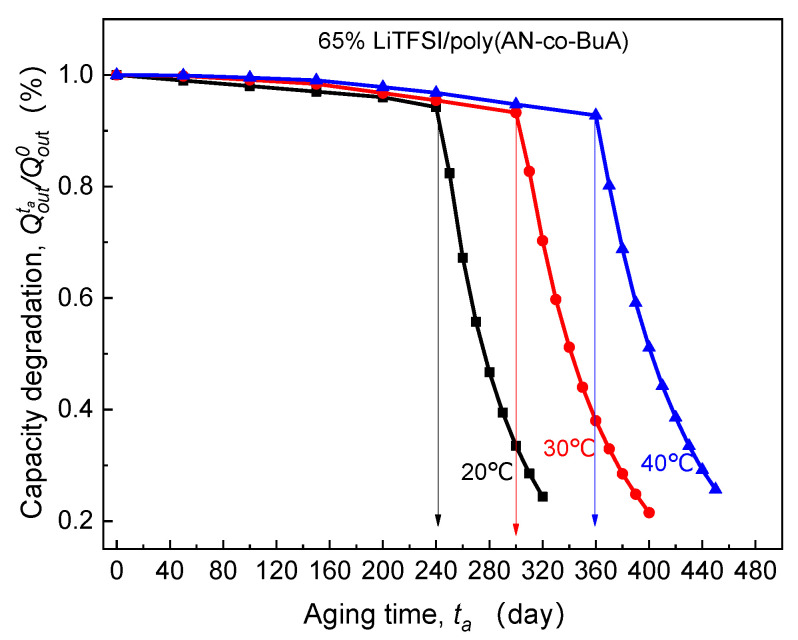
Effect of operating temperature on electrolyte-aging induced capacity degradation.

Generally, the ion transport inside the SPE depends on the thermal movement of the main and side chains [35,36], meaning that the operating temperature is a critical environmental factor influencing the ion diffusion rate and conductivity. Thus it is inevitably related to the aforesaid EICD effect. Under the different environmental temperatures, including 20 °C, 30 °C, and 40 °C, the calculated curves of $Q_{out}^{t_{a}}/Q_{out}^{0}$-$t_{a}$ for ASSB discharged at 1C rate are as shown in Figure 9.

By observing the curves of $Q_{out}^{t_{a}}/Q_{out}^{0}$-$t_{a}$ in the above Figure 9, it is clear that with the increase of the discharge temperature, the critical aging time ($t_{a}^{cr}$) corresponding to the slump in discharge capacity tends to be significantly extended. Surprisingly, after being stored at 40 °C for 360 days, the cell can display over 90% discharge capacity retention, which is much larger than that at 20 °C and 30 °C. This can be attributed to the fact that higher operating temperature increases the vibration of the polar segment near the main chain and augment the free volume fraction of the polymer system [5,37]. Furthermore, it promotes Li^+^ to move from the original coordination region to the other end of the chain through complexation and dissociation. The published experimental results also verified that the temperature-dependent diffusion coefficient of lithium-ion in 65%LiTFSI/poly(AN-co-BuA) electrolyte conforms to the VTF equation [8]. To sum up, appropriately increasing the service temperature may visibly inhibit the capacity degradation caused by the time-aging of SPE and substantially improve the long-term electrochemical performance of the ASSB.

### 3.3. Effect of Lithium Salt Content of SPE

**Figure 10:**
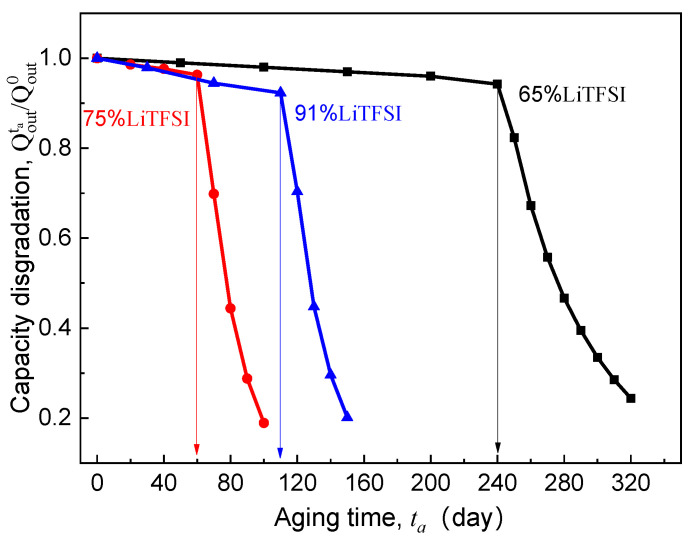
Effect of lithium salt content of SPE on electrolyte-aging induced capacity degradation.

As the carrier of ionic conduction, lithium salt is also of great importance because its content directly determines the number of mobile ions in the SPE. As discussed above, the essence of the cell capacity loss induced by electrolyte aging is that the number of lithium ions flowing into the electrode cannot meet the kinetics of the electrochemical reaction at the electrode surface. This makes lithium salt concentration also a major internal cause of the EICD effect. Based on the experimental ionic conductivity of LiTFSI/poly(AN-co-BuA) by Lasinska et al. [8] (see Figure 2), the following paragraphs will discuss the discharge capacity of the ASSB based on aging electrolyte and compare the corresponding critical aging time when the LiTFSI concentration is set at 65%, 75%, and 91%, respectively. The discharging is at 20 °C and 1C, and the simulation results are shown in Figure 10.

Previously, many studies demonstrate that when the valid ligand is formed between the lithium salt and polymer segment, the ionic migration can be realized through the process of complexation-decomplexation [7,36,38,39]. In addition, lithium salt also affects the regularity of the polymer molecular chain, which reduces the degree of crystallinity and melting point of the electrolyte material, enhances the thermal motion ability of the side segments, and thus improves the ionic conductivity of the SPE. Therefore, a high lithium salt content is supposed to be more able to inhibit the EICD effect. However, for the ASSB discharged at 1C rate and 20 °C, it can be seen in Figure 10 that the critical aging time ($t_{a}^{cr}$) when the $Q_{out}^{t_{a}}/Q_{out}^{0}$ curves steeply drop does not rise monotonously with increasing LiTFSI loading from 65% to 91%. Instead, it has decreased first and then increased as seen in Figure 10. What is the reason behind this phenomenon? Usually, the high-content lithium salt is more likely to form ion clusters or ion pairs, which may reduce the valid ion-carrier concentration and result in a fast decline in ionic conductivity of the electrolyte. By comparing those as mentioned above aging time-dependent ionic conductivity (κ) of LiTFSI/poly(AN-co-BuA) electrolyte with different lithium salt concentrations (see Figure 2), it can be found that the κ value of the electrolyte with 75% LiTFSI is lower than that of the electrolyte with 65% LiTFSI. This is due to the fact that the electrolytes with lithium salt concentrations of both 65% and 75% belong to a metastable system, where the high-content salt produces a larger amorphous salt domain, increasing the distance between polymer chains and reducing the migration probability of lithium ions among polar segments [8]. Besides, the relaxation of side-chain movement in the time-aging process further retards the Li^+^ diffusion leading to an untimely attenuation in the discharge capacity of the ASSB. When the lithium salt content is increased up to 91%, the electrolyte material transforms from “salt soluble in polymer” to “polymer soluble in salt.” The extremely high ion-carrier concentration greatly enlarges the initial ionic conductivity of the electrolyte (as shown in Figure 2). However, the higher free volume fraction makes the thermodynamic state of 91% LiTFSI/poly(AN-co-BuA) electrolyte to be of highly non-equilibrium. Therefore, in the process of time-aging, the decline in κ value of the SPE with 91% LiTFSI is more than those with the other lithium salt concentrations. The discharge capacity of the ASSB with 91% LiTFSI/poly(AN-co-BuA) electrolyte experiences a relatively slow degradation compared with the cell composed of 75% LiTFSI/poly(AN-co-BuA). However, its failure may occur significantly earlier than the ASSB with 65% LiTFSI/poly(AN-co-BuA), and the critical aging time ($t_{a}^{cr}=110$ days) is less than half of the latter.

Moreover, the above analysis may be utilized further to estimate the critical ionic conductivity of the solid electrolyte when the EICD takes place. By comparing the inflection points of $Q_{out}^{t_{a}}/Q_{out}^{0}$-$t_{a}$, curve is shown in Figure 10 and the $\kappa -t_{a}$ curve in Figure 2, it may be concluded that when the ionic conductivity of the LiTFSI/poly(AN-co-BuA) electrolyte is reduced to about 10^−5^ S/cm during aging, the Li^+^ flow that transports across the electrolyte will be insufficient to maintain the lithiation reaction of active material. Thence, to eliminate the EICD effect, it is suggested that the electrolyte conduction should be larger than a critical value within the service life of the ASSB. Some possible methods may be tailoring the electrolyte composition, optimizing the initial ionic conductance, and adjusting the forming process to adapt a proper time-aging rate of the SPE.

### 3.4. Effect of Electrolyte Thickness

**Figure 11:**
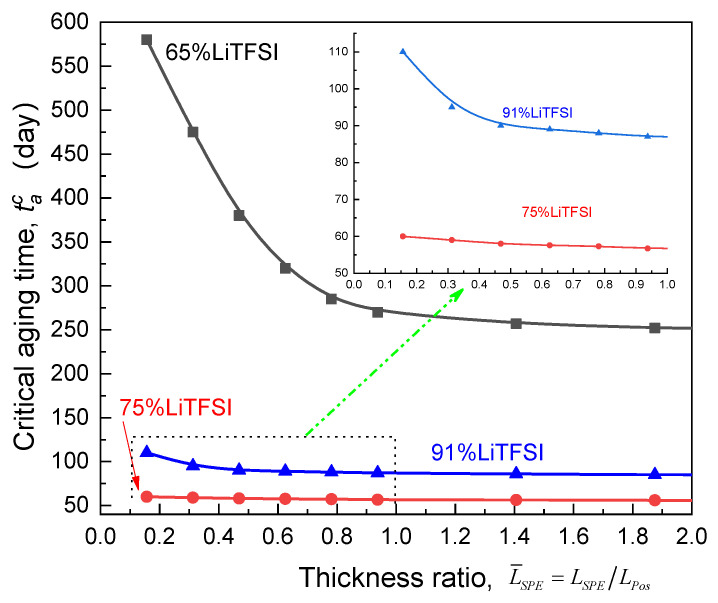
Effect of thickness ratio of the electrolyte relative to the cathode on the critical aging time.

Based on the calculated $Q_{out}^{t_{a}}/Q_{out}^{0}$-$t_{a}$ curves for the ASSB with the SPE films of different relative thickness compared to the cathode, Figure 11 depicts the impact of the thickness ratio (${\overset{¯}{L}}_{SPE}=L_{SPE}/L_{Pos}$) of electrolyte to the electrode on the critical aging time ($t_{a}^{cr}$). Clearly, with regard to the Li/65%LiTFSI/poly(AN-co-BuA)/LiCoO_2_, it can be found that the magnitude of $t_{a}^{cr}$ drops sharply as the relative thickness of electrolyte increases, reaches to an inflection point ${\overset{¯}{L}}_{SPE}^{cr}$ (nearly 1.0) at which $dt_{a}^{cr}/d{\overset{¯}{L}}_{SPE}$ approaches zero and tends to a platform indicating that decreasing the electrolyte thickness helps to relieve the EICD effect. Importantly, the cell of Li/65%LiTFSI/poly(AN-co-BuA)/LiCoO_2_ with the thinner electrolyte film (${\overset{¯}{L}}_{SPE}\leq 0.2$) can keep the stability of discharging capacity during one and a half years, which may meet the demanding requirements for some engineering applications. As the lithium salt concentration of SPE increases from 65% to 91%, raising electrolyte thickness would cause a similar evolution of critical aging time. Further, it is found that the ${\overset{¯}{L}}_{SPE}^{cr}$ of SPE is ranked as follows: 65%LiTFSI > 91%LiTFSI > 75%LiTFSI.

## 4. Conclusions

Accounting for the SPE time-aging during the storage condition, a full-field coupling model of thermodynamics-electrochemistry-mechanics has been established for the ASSB. The cell capacity degradation induced by electrolyte aging is computationally simulated and numerically optimized in detail. Beyond that, the influences of discharge rate, operating temperature, and lithium salt concentration, as well as the electrolyte thickness on this aging effect, are systematically investigated. The main conclusions are as follows.

(1) The time-aging of SPE may fade the electrochemical performance of the ASSB, and there is a critical aging time ($t_{a}^{cr}$) for the slump of discharge capacity. The contact area of the solid electrolyte/electrode interface and the diffusion of Li^+^ inside the electrolyte materials are considered as two pivotal factors controlling the cell decay. In the early stage of aging ($\leq t_{a}^{cr}$), the electrolyte-aging induced capacity degradation (EICD) is dominated by the loss of interfacial contact area. Subsequently, it is controlled by the rate of Li^+^ migration through the SPE.

(2) The essence of the occurrence of EICD is whether the lithium ions flow across the electrolyte and move to the interface with the cathode can satisfy the lithiation reaction of the active materials. The cell service conditions are closely associated with this effect. To be specific, increasing the working temperature may improve the durability of the ASSB, while high-rate discharges would lead to earlier failure.

(3) The impact of lithium salt concentration in the electrolyte on EICD is complicated. In general, increasing the addition of lithium salt may contribute to the extension of the cycle life of the ASSB, while adding excessive lithium salt would accelerate the time-aging process of the SPE and result in the premature deterioration of the cell discharge capacity.

(4) During the service of the ASSB, it is vital to find that the negative effect of electrolyte aging can be restrained by maintaining the ionic conductivity of the SPE above a critical value. Also, a thinner electrolyte film is a benefit for the durability of the ASSB with time-aging SPE.

## Figures and Tables

**Table 1:** State variables and initial and boundary conditions (BCs) for the electrochemical model.

Domains	SPE	Positive Electrode
State variables	$c_{Li^{+}}$, $c_{X^{-}}$, $\phi_{SPE}$	$c_{Li}$
Initial conditions	$c_{Li^{+}}=c_{X^{-}}=\delta c_{Li^{+},total}$, $\phi_{SPE}=0$	$c_{Li}=c_{Li}^{0}$
BCs at *x* = 0	$h_{X^{-}}\cdot n=0$, $h_{Li^{+}}\cdot n=j\cdot n=\;I_{Li}/\left(FA\right)$	
BCs at *x* = *L*_SPE_	$h_{X^{-}}\cdot n=0$, $h_{Li^{+}}\cdot n=j\cdot n=\;j_{Li}\cdot n=I_{LiCoO_{2}}/\left(FA\right)$	
BCs at *x* = *L*_SPE_ + *L*_Pos_	$j_{Li}\cdot n=0$	

**Table 2:** Sets of material parameters used in the simulation.

**Symbol**	**Description**	**Value**
δ	Fraction of free Li ions in equilibrium	0.18 [17,23]
$D_{\mathrm{Li}}$	Diffusion coefficient for Li, positive electrode	1.76 × 10^−15^ m^2^/s [17,23]
$D_{Li^{+}},\;D_{X^{-}}$	Ionic diffusion coefficients of Li^+^ and TFSI^−^	$D_{Li^{+}}=0.2D_{X^{-}}$ [30]
$c_{\mathrm{Li},max}$	Selected maximal concentration of Li, positive electrode	2.33 × 10^4^ $\mathrm{mol}/m^{3}$ [17,23]
$k_{\mathrm{pos}}$	Rate constant charge transfer reaction, positive electrode	5.1 × 10^−4^ $\mathrm{mol}/\left(m^{2}s\right)$ [17]
$k_{\mathrm{neg}}$	Rate constant charge transfer reaction, negative electrode	1 × 10^−2^ $\;\mathrm{mol}/\left(m^{2}s\right)$ [17,23]
$\alpha_{\mathrm{pos}}$	Charge transfer coefficient in positive electrode	0.6 [17,23]
$\alpha_{\mathrm{neg}}$	Charge transfer coefficient in negative electrode	0.5 [23]

## Data Availability

Not applicable.

## Appendix A

The exchange current $I_{Li}^{0}$ and $I_{LiCoO_{2}}^{0}$ in Equation (1) are given by

(A1) $$I_{Li}^{0}=FAk_{Li}^{s}{\left(c_{Li^{+}}/c_{Li^{+},total}\right)}^{\alpha_{Li}}$$

(A2) $$I_{LiCoO_{2}}^{0}=FAk_{LiCoO_{2}}^{s}{\left[\frac{(c_{Li,max}-\;c_{Li}\;)c_{Li^{+}}}{\left(c_{Li,max}-c_{Li,min}\right)c_{Li^{+},total}}\right]}^{\alpha_{LiCoO_{2}}}{\left(\frac{\;c_{Li}-c_{Li,max}\;}{c_{Li,max}-c_{Li,min}}\right)}^{1-\alpha_{LiCoO_{2}}}$$

where $k_{i}^{s}$($i=Li,\;LiCoO_{2}$), $\alpha_{i}$($i=Li,\;LiCoO_{2}$), and $c_{Li^{+}}$ are the reaction rate constant, the charge transfer coefficient, and the concentration of mobile Li-ions, respectively. Meanwhile, $c_{Li^{+},total}$, $c_{Li,max}$, and $c_{Li,min}$ are the total concentration of Li ions in the solid electrolyte, the maximum and minimum lithium concentration in the positive electrode, respectively.

## Appendix B

The hydrostatic pressure *p* is determined by the following governing Equations of elastic mechanics, which include the equilibrium equation, i.e.,

(A3) ∇⋅σ=0,

the strain-displacement equation, i.e.,

(A4) $$\varepsilon =\frac{1}{2}\left(\nabla u+u\nabla \right),$$

and the constitutive equation, i.e.,

(A5) σ=2Gdevε−pI,

where σ, ε, and u are the stress, the strain, and the displacement tensor, respectively. Besides, the volume modulus and the shear modulus are represented by the symbols K and G.
